# From Microbiome to Malignancy: Unveiling the Gut Microbiome Dynamics in Pancreatic Carcinogenesis

**DOI:** 10.3390/ijms26073112

**Published:** 2025-03-28

**Authors:** Dhanisha Sulekha Suresh, Tejeshwar Jain, Vivaan Dudeja, Srikanth Iyer, Vikas Dudeja

**Affiliations:** Division of Surgical Oncology, Department of Surgery, The University of Alabama at Birmingham, BDB 573 1808 7th Avenue South, Birmingham, AL 35294, USA; dsulekhasuresh@uabmc.edu (D.S.S.); tjain@uabmc.edu (T.J.); vivaandudeja@gmail.com (V.D.); srikanthiyer@uabmc.edu (S.I.)

**Keywords:** pancreatic cancer, gut microbiota, metabolites, bile acids, oncobiome

## Abstract

Pancreatic cancer is a major cause of cancer-associated mortality globally, characterized by a poor prognosis and limited therapeutic response. The current approach for treating pancreatic cancer involves locoregional control with surgical resection and systemic therapy in the form of cytotoxic chemotherapy. However, despite standard-of-care treatment, the outcomes remain dismal. Emerging evidence suggests that the gut microbiota plays a significant role in pancreatic carcinogenesis through dysbiosis, chronic inflammation and immune modulation. Dysbiosis-driven alterations in the gut microbiota composition can disrupt intestinal homeostasis, promote systemic inflammation and create a tumor-permissive microenvironment in the pancreas. Moreover, the gut microbiota modulates the efficacy of systemic therapies, including chemotherapy and immunotherapy, by impacting drug metabolism and shaping the tumor immune landscape. This review is mainly focused on exploring the intricate interplay between the gut microbiota and pancreatic cancer, and also highlighting its dual role in carcinogenesis and the therapeutic response.

## 1. Introduction

Pancreatic cancer is an extremely challenging and aggressive cancer, characterized by poor outcomes and limited treatment options. The GLOBOCON 2020 report indicates that the incidence differs among nations, often showing an increased pattern in developed countries compared to others [1]. Pancreatic cancer can develop from either the endocrine or exocrine regions of the pancreas. Pancreatic ductal adenocarcinoma (PDAC), arising from the exocrine pancreas, is the most prevalent malignancy of the pancreas, comprising around 90% of all cases [2]. PDAC has emerged as a significant cause of cancer-related mortality in the United States, now ranking as the third leading cause of cancer deaths, and is predicted to surpass colorectal cancer by 2040. Strikingly, PDAC has an almost equal incidence-to-mortality ratio, signifying its poor prognosis and resistance to existing therapies. Surgical resection is the only curative option; however, fewer than 20% patients present with resectable disease. Patients who undergo successful resection have a significantly better survival than patients with unresectable disease [2]. This underscores the critical importance of the early detection and accurate diagnosis of pancreatic cancer in improving the rate of curative surgeries for pancreatic cancer. Carbohydrate antigen (CA) 19-9 is the only serum biomarker currently utilized in the clinical management of pancreatic cancer, with elevated levels observed in approximately 80% of cases. However, it is limited by its lack of specificity as it can be elevated in other biliary pathologies like cholangitis and extrapancreatic malignancies. Additionally, 5–10% of the population do not secrete CA 19-9 and therefore, these undersecretors are unable to be detected by CA 19-9 [3]. There has been extensive research into other biomarkers such as miRNAs, circulating tumor cells (CTCs), ctDNA and mucins; however, none of these have been incorporated into the diagnostic algorithm for PDAC [4]. Thus, finding biomarkers for the early diagnosis of PDAC remains an area of active investigation. Another challenge in the management of PDAC is the high resistance of cytotoxic chemotherapies, which are used for patients presenting with locally advanced or metastatic disease [5]. This is attributed to its dense desmoplastic stroma, poor vascularity and immunologically “cold” tumor microenvironment (TME). In addition to chemotherapy, PDAC is notoriously resistant to immunotherapy such as immune checkpoint blockade (ICB), which has been successful in multiple hematological and solid-organ malignancies [6]. Therefore, there is an urgent need to identify therapeutic targets to improve the response to systemic therapies and, consequently, improve patient outcomes.

The human gut microbiota comprises over 10^14^ microbes, representing a diverse array of more than 1000 species. The gut microbiome primarily comprises several major phyla, notably *Bacteroidetes*, *Firmicutes*, *Proteobacteria*, *Actinobacteria*, *Verrucomicrobia* and *Fusobacteria.* Among these, *Firmicutes* and *Bacteroidetes* are the most prevalent, collectively accounting for approximately 90% of the microbial population [7]. These intestinal microbes significantly impact human physiology by modulating the immune system, affecting metabolism, maintaining intestinal structural integrity, providing nutritional support, balancing hormones and facilitating digestion by breaking down complex fats, proteins and carbohydrates that are otherwise indigestible by the host [8]. In addition, the gut microbiome also helps to synthesize essential vitamins, including vitamin K and vitamin B (folate, biotin and B12), and produces bioactive metabolites essential for regulating host metabolic processes. In addition, microbes within the gastrointestinal tract can metabolize the polysaccharides into short-chain fatty acids (SCFAs) (butyrate, propionate and acetate) and monosaccharides [8,9,10]. Microbial composition varies among individuals, shaped by factors such as lifestyle, diet, ethnicity, genetic makeup and medications [11]. Although commensal bacteria are fundamental to maintaining homeostasis across various physiological processes, disruptions in gut microbiota composition and function, termed “dysbiosis”, have emerged as a key factor in the pathogenesis of various human diseases including liver cirrhosis, inflammatory bowel disease, non-alcoholic fatty liver disease and multiple malignancies [12].

In context of the pancreas, there is accumulating evidence to indicate the existence of a gut–pancreas axis that functions during homeostasis as well as in pathological states. The pancreas secretes numerous proteins such as α-defensins, Reg proteins and GP2 that play a protective role against invasive pathobionts in the intestine and help to maintain the commensal microbiome [13]. The gut microbes themselves have also been shown to affect the pancreatic environment. Gut microbes can translocate to the pancreas via the bloodstream or the mesenteric lymph nodes and affect disease processes such as acute pancreatitis, diabetes and pancreatic cancer [14]. Indeed, patients with inflammatory bowel disease (IBD), who are known to possess a dysbiotic microbiome, are at an increased risk of pancreatic diseases including pancreatitis and pancreatic cancer [15]. Biliary reflux into the pancreatic duct is another modality for microbial colonization of the pancreas, and patients undergoing biliary instrumentation, such as ERCP, have been shown to have a higher bacterial abundance in their pancreatic secretions [16].

**Figure 1:**
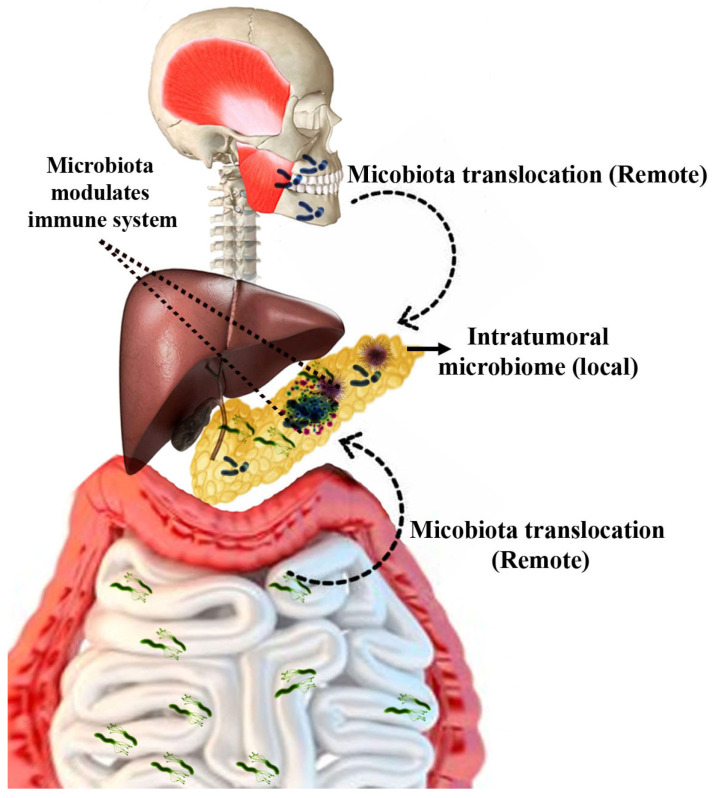
The impact of microorganisms on pancreatic cancer progression remotely and locally. Gut microbiota and oral microbiota can inhibit the antitumor immunity of host, aiding in the progression of PDAC. Similarly, intrapancreatic microorganisms (local) also contribute to pancreatic carcinogenesis.

The term “oncobiome” describes the research on the impact of the microbiome on human cancer development [17]. Accumulating evidence now strongly supports the involvement of microbes in different malignancies, including pancreatic cancer. Microorganisms may affect pancreatic cancer progression via local or systemic mechanisms (Figure 1) [18]. Locally, microbes can influence tumor progression by modulating immune responses, inducing inflammatory pathways and altering cellular metabolism. In the context of the pancreas, which was traditionally considered a sterile organ, multiple studies have demonstrated the presence of microbial flora. Preclinical studies indicate that these microbes can directly interact with the tumor microenvironment and even affect the metabolism of chemotherapeutic drugs in the pancreas [17]. On the systemic level, microbial products such as metabolites or endotoxins, secreted into the circulation, can affect the systemic antitumor response or reach the TME via the bloodstream and modulate tumor growth. Interestingly, some of the established PDAC risk factors, including smoking [19], alcohol consumption and obesity [20,21], are known to affect the gut microbiome. Moreover, correlational studies indicate that patients with PDAC harbor a distinct gut microbiome signature [22]. In summary, the gut microbiota influences the initiation and progression of PDAC; however, the precise mechanisms underlying this complex interaction remain unclear, emphasizing the need to further explore the microbiome’s involvement in PDAC. This review highlights recent progress in microbiome research related to PDAC and also identifies the existing knowledge gaps and proposes future directions to address the challenges comprehensively. This review is based on a thorough analysis of peer-reviewed articles and research papers sourced from reputable databases, including Scopus and PubMed. Relevant studies were identified using keywords such as “pancreatic cancer”, “gut microbiome”, “microbiota and carcinogenesis” and “immune response in pancreatic tumors”. Articles were selected based on the relationship between the gut microbiota and pancreatic carcinogenesis, emphasizing recent findings and advances highlighting the role of the microbiome in tumor progression and therapeutic implications.

### 1.1. Gut Microbiome Signature in PDAC—Potential as an Early Biomarker?

Despite advances in diagnostic techniques, including imaging, serum markers and the identification of high-risk pre-malignant lesions, the early detection of PDAC remains a significant challenge. There is a critical need for non-invasive and highly accurate biomarkers to expand screening and facilitate earlier detection and intervention. Microbial profiling revealed distinct alterations in both phylum and genus levels in PDAC patients compared to healthy individuals. Thus, this microbial shift could serve as a non-invasive tool for the early detection and better classification of PDAC cases [23].

A study by Kartal et al. highlighted the potential of the gut microbiome as a highly specific, non-invasive tool for the early detection of PDAC. Their analysis revealed that fecal metagenomic classifiers were more effective than saliva-based methods, achieving an impressive area under the AUROC (receiver operating characteristic curve) of up to 0.84. Remarkably, this accuracy was consistent across both the early and late stages of PDAC, driven by a distinctive panel of 27 microbial species. Notably, a microbiota-based classification model centered on PDAC-enriched species showed exceptional disease specificity. Additionally, the integration of microbiome-based predictions with CA-19-9—currently the only FDA-approved non-invasive biomarker for PDAC—demonstrated a substantial improvement in diagnostic accuracy. The combined approach achieved an AUROC of up to 0.94, highlighting the potential of microbiome profiling to enhance the sensitivity and specificity of PDAC detection. When validated against 25 publicly available metagenomics datasets encompassing a wide range of health conditions (*n* = 5792), the model consistently maintained its accuracy and specificity. Additionally, the microbiome-based models exhibited a strong predictive performance when validated with an independent German cohort (*n* = 76) [24]. Nagata et al. conducted a similar cross-sectional study where metagenomic analysis was performed on fecal and salivary samples from patients with PDAC and healthy controls, as well as patients with IPMN and CP [25]. The discovery cohort consisted of 47 Japanese PDAC patients and 235 matched healthy controls. The authors used extensive matching to account for confounders including age, sex, body mass index, lifestyle-related factors, dietary habits, dental/oral problems, comorbidities, bowel diseases, Bristol stool scale and medications. They found 30 gut and 18 oral species that were significantly altered in the PDAC cohort. Using these species as classifiers, the study achieved a high diagnostic accuracy, with AUC values of 0.78 for the gut microbes and 0.82 for oral microbes in distinguishing PDAC from controls. However, when the authors used these classifiers in validation cohorts consisting of samples collected in Spain and Germany, the gut microbiome-based classifier outperformed the oral microbiome-based classifier in both cohorts. Notably, a consistent microbial signature observed across all three cohorts included an enrichment of *Veillonella* and *Streptococcus* species, along with a depletion of *Faecalibacterium prausnitzii* in the gut microbiome [25]. The identification of shared gut microbial signatures in European and Asian cohorts underscores the existence of globally conserved microbiome-based biomarkers for PDAC detection and risk stratification. In addition, a recent meta-analysis involving 1511 individuals, including PDAC (*n* = 285), chronic pancreatitis (CP) (*n* = 342) and healthy controls (*n* = 649), revealed a significant alteration in gut microbiota across PDAC and CP patients compared to healthy controls [26]. Both groups exhibited a slight reduction in α-diversity and a significant difference in β-diversity as compared to healthy controls, with an enrichment of pathogenic bacteria like *Veillonella* and *Escherichia-Shigella.* However, no significant difference was observed between PDAC and CP patients, suggesting a common pattern [26]. Given the variability in 16s library preparation and analysis, as well as the inability to account for confounders such as diet, geographical location, disease stage, etc., the findings should be interpreted with caution.

### 1.2. Other Correlative Studies Between Microbial Dysbiosis and PDAC

Apart from the gut microbiome, microbial dysbiosis in various other locations such as the duodenum, oral cavity and pancreas have also been linked to PDAC development and progression. Historically, the pancreas was considered a sterile organ, but recent studies have identified bacterial populations in both normal pancreas and PDAC samples. The pancreas is linked to the gastrointestinal tract via the pancreatic duct, facilitating the translocation of gut microbiota. It has been speculated that bacterial reflux from the biliary or duodenal tract causes the colonization of gut microbiota within the pancreas [27]. In a limited case–control study, the duodenal mucosa of 14 patients with PDAC and 14 healthy individuals was analyzed by LEfSe (linear discriminant analysis effect size) at the genus level, revealing a significant enrichment of bacterial genera in the duodenal mucosa of PDAC patients, including *Aquabacterium*, *Acinetobacter*, *Rahnella*, *Oceanobacillus*, *Delftia*, *Massilia*, *Sphingobium* and *Deinococcus*. In contrast, the duodenal microbiota of healthy individuals was characterized by a higher abundance of general such as *Paenibacillus*, *Porphyromonas*, *Escherichia*, *Enhydrobacter*, *Pseudomonas*, *Shigella* and *Escherichia* [28]. Further, a subsequent investigation exploring the microbial composition of duodenal fluid during a secretin stimulation test of around 98 individuals diagnosed with pancreatic cysts, 74 samples from PDAC patients and 134 healthy controls revealed a marked increase in bacterial populations, particularly *Rothia*, *Bifidobacteria* and *Fusobacteria,* in PDAC patients; conversely, patients with pancreatic cysts exhibited greater fungal diversity, especially *Saccharomyces* and *Ascomycota,* compared to healthy controls,. However, no significant differences were observed in the duodenal fluid bacterial profile between patients with pancreatic cysts and healthy controls [29]. 

In a seminal study, Nejman et al. analyzed the bacterial population of 1010 different tumor samples and 516 normal adjacent tissue samples, encompassing seven common tumor types, including PDAC. Using a combination of FISH and immunohistochemistry, they were able to detect intratumoral bacteria inside the epithelial and immune compartments. Notably, they observed that a majority of the pancreatic cancer specimens had detectable bacterial DNA, with the most prevalent bacteria belonging to the *Proteobacteria* phylum [30]. Geller et al. analyzed the presence of bacteria by 16s rRNA analysis in around 20 normal pancreatic samples and 113 PDAC samples and were able to detect an increased prevalence of bacteria belonging to the family *Pseudomonadaceae* and *Enterobacteriaceae* in PDAC specimens [31]. Similarly, Pushalkar et al. compared the bacterial composition between PDAC and the normal pancreas. They found that the relative abundance of microbes in the PDAC samples was higher compared to the normal pancreas. Sequencing studies revealed that the most prevalent intratumor phyla comprised *Bacteroidetes*, *Proteobacteria* and *Firmicutes* [32]. Cumulatively, we can conclude that pancreatic tumor tissue has a unique bacterial signature compared to the normal pancreas. The specific microbial populations, which are notably enriched in PDAC, may play a critical role in tumor progression and resistance to therapy. By identifying and profiling these microbial signatures, we can enhance diagnostic accuracy, improve prognostic predictions and also develop personalize therapeutic approaches.

Apart from the intratumoral microbiome, the oral microbiome has also been linked to pancreatic carcinogenesis. The human oral cavity harbors by more than 700 microbial species, which typically maintain a relatively stable population in healthy individuals [33]. Poor oral hygiene and associated conditions such as tooth loss and periodontal disease have been identified as risk factors for PDAC. In a 16-year health follow-up analysis involving around 51,529 American men with periodontal disease, Michaud et al., identified around 216 patients who developed PDAC. Individuals diagnosed with periodontal disease showed a 64% higher risk of PDAC compared to those without it. *Porphyromonas gingivalis* emerged as the prevailing bacterium in the oral cavity of this cohort [34]. Patients diagnosed with both PDAC and periodontal disease show elevated levels of antibodies against *Porphyromonas gingivalis,* at least two-times higher than those seen in healthy individuals [35]. Fan et al. examined the oral microbiome of 361 patients with incident PDAC and 371 matched healthy controls from prospectively maintained databases from the American Cancer Society Cancer Prevention Study II and the National Cancer Institute Prostate, Lung, Colorectal and Ovarian Cancer Screening Trial [36]. They found an increased prevalence of *Aggregatibacter actinomycetemcomitans* and *Porphyromonas gingivalis* along with decreased levels of *Leptotrichia* in patients with PDAC. Thus, it appears that oral dysbiosis is intimately linked to the development of PDAC. However, more mechanistic studies are required to conclusively establish a causative link [36]. Table 1 summarizes some of the available evidence regarding microbial dysbiosis and PDAC.

### 1.3. Mechanism—Gut Microbiota in the Progression of Pancreatic Cancer

The complex mechanisms through which microorganisms influence tumor pathogenesis and progression can be divided into three main categories: how microbes, particularly bacteria, modulate the host immune system and sustain cancer-associated inflammation; how the microbial metabolites and toxins affect the host metabolism; and how the microbiota influences the tumor microenvironment of PDAC. The gut microbiome associated with cancer can elicit innate and adaptive immune responses, ultimately leading to evasion and immune suppression [39,40,41,42,43,44,45,46,47,48]. Generally, microbial dysbiosis in cancer patients disrupts the delicate equilibrium of the gut microbiome, leading to chronic activation of the innate immune system. Paradoxically, instead of protecting the host, this inflammation can foster a tumor-promoting microenvironment by supporting angiogenesis, recruiting MDSCs and suppressing effective antitumor immunity, thereby enabling cancer progression [49,50]

With regards to PDAC, Sethi et al. provided compelling evidence that the gut microbiome plays a critical role in tumor progression by modulating immune responses in rodents. This groundbreaking research explored how antibiotic therapy affects the progression of various cancers in murine models. Remarkably, the administration of an oral antibiotic regimen consisting of neomycin, vancomycin, metronidazole, ampicillin and amphotericin B significantly curbed tumor growth. In addition, this intervention also markedly reduced the tumor burden in a liver metastasis model. However, these tumor-suppressing effects vanished in RAG1 knockout mice, which lack functional T and B cells, underscoring the essential role of immune cells in mediating microbiota-driven tumor suppression. Delving deeper, this study revealed that the tumor-suppressive effects of oral antibiotics were associated with an increased infiltration of Tc1 (IFN+CD8+CD3+) and Th1 (IFN+CD4+CD3+) cells within the tumor microenvironment. Using the IL-17 depletion strategy, the authors concluded that the effects of the gut microbiome on the immune TME of PDAC were being mediated through IL-17. This is an interesting observation as IL-17 inhibitors are in clinical use for autoimmune conditions where IL-17 leads to pathogenic inflammation. However, it seems that the role of IL-17 is context-dependent, as it appears to be an immunosuppressive moiety in PDAC TME [51]. This theory is supported by the work of other authors as well. For example, studies by Mucciolo et al. and Picard et al. recently found that IL-17 plays a very crucial role in driving PDAC progression. It facilitates the recruitment of immunosuppressive granulocytes into the TME, while simultaneously maintaining the stem-like properties of cancer cells, highlighting its dual role in shaping tumor growth and immune evasion [52,53]. Pushalkar et al. demonstrated that gut microbiota depletion significantly reduces myeloid-derived suppressor cell (MDSC) infiltration and reprograms tumor-associated macrophages (TAMs) towards an M1-like phenotype. In addition, specifically, gut bacterial ablation enhances the Th1 polarization of CD4+ T cells and also augments the cytotoxic efficacy of CD8+ T cells, characterized by the increased expression of T-BET (a transcription factor that drives the differentiation of CD8+ T cells), TNFα and IFN-γ. In addition, TNFα and IFN-γ produced by CD8+ T cells help to eliminate tumor cells by inducing apoptosis and inhibiting tumor growth. This profound reprogramming of the tumor microenvironment coupled with elevated PD1 expression on effector T cells suggests that combining oral therapeutics with checkpoint-directed immunotherapy is a powerful experimental strategy for PDAC treatment [32]. In contrast to the findings of Sethi et al. and Pushalkar et al., where the gut microbiome seems to interact with the adaptive and innate immune system and affect PDAC growth, Thomas et al. reported that gut microbiome depletion was able to abrogate pancreatic xenograft growth in Nod-Scid mice that lack an adaptive immune system, thus arguing for an effect independent of the adaptive immune system [54]. More recently, Yu et al. demonstrated that microbial-derived components play a pivotal role in modulating natural killer (NK)-cell-mediated antitumor immunity, thereby influencing the progression of PDAC [55].

A study by Aykut et al. highlighted the significant role of mycobiota in the progression of PDAC. Their analysis revealed that fungi translocate from the gut to the pancreas and accelerate tumor progression through the activation of a mannose-binding lectin (MBL)-C3 cascade. In addition, they also observed that the mycobiome composition in PDAC was markedly distinct from that of the normal pancreas or gut. Notably, the ablation of mycobiota significantly suppressed tumor progression, whereas the reintroduction of the fungal species *Malassezia* accelerated oncogenesis [37]. Similarly, a study by Alam et al. also highlighted the pivotal role of mycobiota in the progression of PDAC. While it is well established that Th2 and innate lymphoid cells can promote tumor progression by secreting pro-inflammatory cytokines such as interleukin (IL)13, IL4 and IL5, the underlying mechanism driving this phenomenon in PDAC has remained unclear. Alam et al. shed light on this by demonstrating that the oncogenic KRAS mutation induces the expression of IL33, which serves to recruit and activate Th2 cells and innate lymphoid cells, fostering a pro-tumorigenic immune environment. Strikingly, the tumor-specific deletion of IL33 resulted in significant tumor regression. Further, they identified the intratumoral fungal mycobiome as the source of IL33 in PDAC. Furthermore, antifungal treatment not only reduced IL33 expression but also resulted in tumor regression, highlighting a novel interplay between fungal mycobiota and immune pathways in PDAC progression [56].

Although a plethora of preclinical studies now indicate that both bacterial and fungal commensal species can influence PDAC inception and progression, more stringent studies are needed to prove whether this association truly exists in the context of human PDAC.

### 1.4. Effect of Gut Microbiome on Therapy Response in PDAC

**Figure 2:**
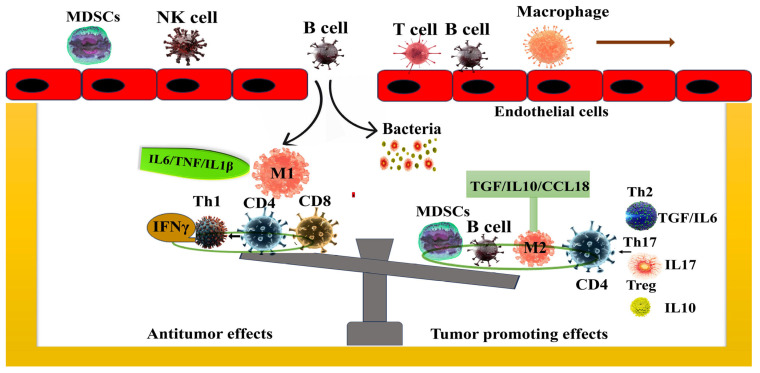
The microbiota shapes the immune microenvironment of the tumor: Within the TME (tumor microenvironment), the microbiota triggers the immune system and attracts immune cells. Under the influence of the microbiota, these immune cells undergo differentiation into distinct immune cell subtypes that secrete specific factors which exert either anti- or pro-neoplastic effects. For example, the presence of microbiota could lead to a decrease in immune cells known for their antitumor effects, such as CD8+ T cells, M1 macrophages (releasing IL-β/IL6/TNF) and Th1 cells (from CD4+ T cells and secreting IFN-γ). Conversely, microbiota could elevate immune cells with pro-tumorigenic effects, which include B cells, M2 macrophages (secreting IL10/TGF/CCL18), MDSCs, and Th2 (CD4+ cells; secreting IL6/TGF), Th17 (secreting IL17) and Treg (secreting IL-10) cells.

A study by Geller et al. has revealed a striking phenomenon by which bacteria can transform the chemotherapeutic agent gemcitabine to its inactive form 2′,2′-diflurodeoxyuridine through the bacterial enzyme cytidine deaminase, predominantly expressed in *Gammaproteobacteria.* This bacterial-driven conversion leads to the development of gemcitabine resistance in colon cancer tumors. This resistance was completely reversed when the antibiotic ciplofloxacin was used in combination. An analysis of human PDAC samples revealed that approximately 76% of 113 human PDAC samples harbor *Gammaproteobacteria*, suggesting a significant prevalence of these bacteria within the PDAC microbiome. Their presence may directly contribute to the chemotherapy resistance, complicating the treatment landscape of this aggressive cancer. Given that *Gammaproteobacteria* predominate in PDAC and mediate gemcitabine resistance, strategies aimed at inhibiting cytidine deaminase (CDD) activity or modulating the microbiome to minimize bacterial-mediated drug resistance could significantly enhance gemcitabine therapy in PDAC. This approach emphasizes the necessity of integrating microbial dynamics into the design of more effective, microbiome-based cancer therapies, ultimately improving treatment outcomes for PDAC patients [31]. Shotgun metagenomic sequencing and metabolic analysis in PDAC have revealed that patients responding to chemotherapy showed higher levels of indole-3-acetic acid (IAA), a microbiota-derived metabolite from tryptophan. Further experimental interventions such as fecal microbiota transplantation, tryptophan administration and dietary supplementation with IAA have demonstrated the ability to enhance chemotherapy’s efficacy in humanized gnotobiotic mouse models of PDAC [57]. The microbiota could alter the ratio of immune cell components within the TME, indirectly impacting the tumor’s immune response and thus modifying its malignant characteristics (Figure 2). This indirect regulation of the tumor’s immune response can influence key processes such as immune surveillance, immune evasion and inflammation, ultimately altering tumor progression, aggressiveness and therapeutic responsiveness.

### 1.5. The Impact of Gut Microbiome on Clinical Outcomes

A small study by Kharofa et al. evaluating 24 PDAC patients who underwent pancreatic resections linked the gut microbiome to survival outcomes. They analyzed stool samples from patients with a recurrence-free survival of >4 yrs (*n* = 16) compared to a second cohort of patients with median disease free survival of 1.8 yrs (*n* = 8). A shotgun metagenomic analysis of stool samples from these LTS patients revealed a striking enrichment of bacterial species within the *Ruminococcaceae* family, particularly *Akkermansia muciniphila* and *Faecalibacterium prausnitzii*, indicating that specific microbial species could be linked to favorable survival outcomes [58]. However, the data should be interpreted with caution due to the small sample size, lack of appropriate controls and retrospective nature of the study. Riquelme et al. [59] provided compelling evidence that the intratumoral microbiome can also differentially affect survival outcomes. In their study, an increased intrapancreatic abundance of the *Pseudoxanthoma*, *Saccharopolyspora*, *Baccilus clausii*, and *Streptomyces* was observed in patients with long-term survival [59]. Moreover, LTS showed an increased expression of antitumor immune markers such as CD8+, CD3+ and Granzyme B+ cells within the tumor, suggesting the intratumor microbiome may promote immune activation within the tumor microenvironment, promoting a more robust antitumor immune response. Interestingly, the authors found that the gut microbiome could affect the intratumoral microbiome composition, indicating that the gut microbiome might indirectly affect PDAC survival outcomes as well [58]. Recent research has unveiled striking differences in the gut microbiota profiles of patients with resectable and unresectable PDAC. Unresectable PDAC patients show a significant depletion of beneficial genera such as *Anaerostipes, Alistipes, Parvimonas* and *Faecalibacterium*, alongside increased levels of *Cloacibacterium, Pseudonocardia, Anaerotruncus and Anaerotruncus* [60]. However, no completed clinical trials have yet explored whether microbiome modulation in unresectable PDAC patients could offer therapeutic benefits. The connection between PDAC and the gut microbiome remains an emerging field and holds promise to redefine therapeutic strategies for unresectable PDAC, transforming a grim prognosis into an opportunity for innovation and hope.

### 1.6. Clinical Trials

Efforts are underway to translate our understanding of the microbiome’s role in PDAC into clinical practice. Several prospective observational studies are exploring the link between gut microbiome and PDAC. For example, multiple studies are ongoing to investigate the changes in the fecal microbiome following gastrointestinal surgery, including pancreatic surgery (NCT04274972, NCT03809247, NCT03840460, NCT3840460, NCT04203459, NCT04638751, and NCT04363983) [61]. However, translation of the accumulating preclinical evidence linking the gut microbiome to PDAC, into interventional studies is challenging. This is evidenced by the withdrawal of two therapeutic interventions studies that were initiated recently (NCT03891979 and NCT04600154). Comprehensive data regarding the reasons for withdrawal are not available; therefore, it is difficult to speculate as to the reasons. There are multiple challenges involved in modulating the gut microbiome. One of the key challenges is that altering one microbial population can lead to compensatory changes in other microbial species, potentially resulting in unintended consequences, such as dysbiosis or the overgrowth of opportunistic bacteria. Additionally, the microbiome consists of a vast array of organisms that vary between individual-based genetics, lifestyle, diet and environmental factors, making targeted intervention difficult. Moreover, the interactions between the microbiome and host immune responses add another layer of complexity, as certain microbial shifts may trigger pro-inflammatory or immunosuppressive effects. Additionally, while probiotics, prebiotics, FMT and antibiotics have been explored as potential strategies of microbiome modulation, their long-term efficacy, safety and reproducibility remain areas of active investigation.

### 1.7. Future Aspects

**Figure 3:**
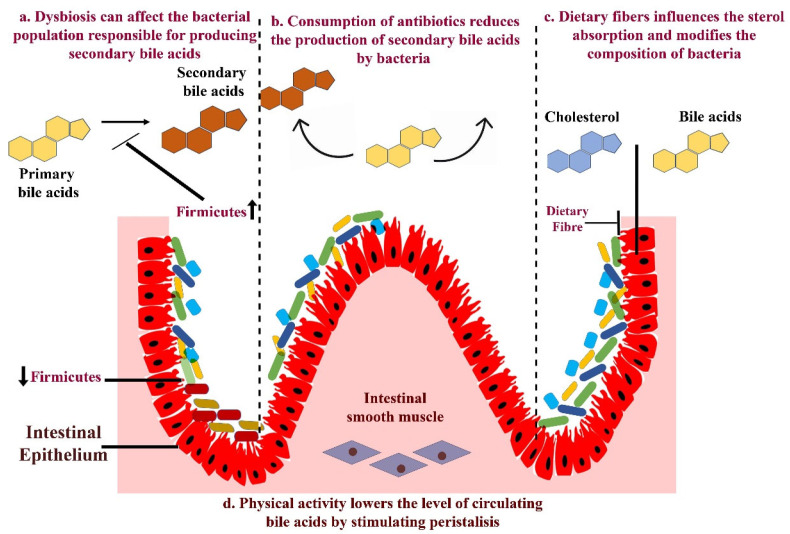
Factors affecting bacterial bile acid metabolism.

There is an urgent need for more extensive studies to unravel the mechanisms underlying the impact of the gut microbiome on both progression and treatment of pancreatic cancer. Subsequently, a patient-centered approach targeting the microbiome could be a promising prospect for advancing PDAC therapeutics. The role of bacteria in cancer progression has been extensively studied in the microbiome due to their predominance [62]. However, the human microbiome encompasses other components such as fungi and viruses, which can also influence disease development in the host [63]. Hence, further research is imperative to enhance our understanding of how commensal fungi and viruses contribute to pancreatic carcinogenesis. Stromal components shape the immune milieu within tumors by paracrine signals, such as metabolic cues and cytokines. Cancer-associated fibroblasts within stroma are recognized as a primary source of tumor resistance. Consequently, therapies targeting stromal markers are currently under investigation in clinical trials to enhance the effectiveness of existing immunotherapies. Considering the available literature, it is noteworthy that there is currently no evidence linking the involvement of the microbiome and PDAC stroma. This aspect is certain to be investigated further as we continue to unravel the microbiome’s impact on tumor development [64,65,66]. Various cancer risk factors such as age, diet, smoking, obesity, alcohol consumption and a high-fat diet have been revealed to induce considerable dysbiosis. It will be interesting to evaluate whether strategies to modulate the gut microbiome can serve as primary prevention measures against cancer. However, this needs to be answered through prospective and epidemiological studies. Further, investigation is necessary to understand the involvement of the gut microbiome in cancer recurrence and metastasis. Extracellular vesicles from microbes can transport metabolites, signaling molecules or antigenic proteins capable of inciting immunomodulation and inflammatory responses in pathological and homeostatic conditions. Furthermore, extracellular vesicles play a crucial role in the development of tumors by facilitating metastasis, delivering proliferative signals, and also helping to promote immune evasion. Pancreatic cancer-derived extracellular vesicles play pivotal roles in orchestrating several critical steps associated with tumorigenesis which include immune evasion, chemoresistance, stromal reprogramming, cancer-associated thrombosis, angiogenesis and proliferation. Due to the limited exploration in this area, it remains elusive whether the extracellular vesicles serve as a mechanism through which the microbiome influences the immune system to affect carcinogenesis [66]. The interaction between the gut microbiota-produced metabolites and the host, and their impact on carcinogenesis, needs to be explored further as well. Metabolites such as bile acids are known to regulate the gut microbiome composition through their antimicrobial properties and modulating host signaling pathways that maintain intestinal homeostasis. Therefore, any disruption in the bile acid levels or composition can lead to a shift in the microbiota, promoting inflammation or creating an environment conducive to opportunistic pathogen infections (Figure 3). Bile acids not only influence the gut microbiota composition but can also alter the functional performance of gut microbiota and significantly influence the progression of inflammation and cancer. Highly hydrophobic secondary bile acids, such as deoxycholic acid (DCA) and lithocholic acid (LCA), are more carcinogenic. They disrupt the intestinal barrier, induce dysbiosis and promote carcinogenesis by generating ROS. Bile acids can also directly affect cancer growth via degrading p53, activating PLA2 and increasing COX-2 in PDAC cells, enhancing cancer cell invasion and underscoring the bile acid–microbiome–cancer axis [67,68,69]. Indeed, gut microbiome-mediated bile acid metabolism has been shown to affect hepatocellular carcinoma growth via regulating NKT cells [45]. Given the multiple roles of bile acids in cancer growth and in maintaining the gut microbiome, it will be interesting to explore this interaction in the context of PDAC growth.

With advancements in technology, extensive metadata are being generated through metabolomics, metagenomic and proteomic studies. By integrating the data from both clinical and preclinical studies and by computational bioinformatics, we have the opportunity to generate a comprehensive reference database, which would encompass functional and phylogenic changes in the microbiome, as well as host-specific responses and disease stages. Such resources could serve as a cornerstone, propelling the next stage of microbiome–cancer research.

### 1.8. Conclusions

The gut microbiome plays a very crucial role in maintaining health and has been increasingly recognized as a key factor in the development and progression of PDAC. Dysbiosis or imbalance in microbiota composition has been implicated in the modulation of the immune response, oncogenesis and patient survival post-resection. Beyond their direct roles, microbial-derived metabolites are gaining attention for their dual role as both mediators of therapeutic efficacy and diagnostic markers. These findings underscore the profound impact of gut microbiota in cancer pathogenesis and its therapeutic potential. Comprehensive understanding of the effect of the gut microbiome could pave the way for the identification of novel biomarkers that not only contribute to the early detection of PDAC, but also provide predictive tools for determining the patient response to specific therapeutic interventions.

## Figures and Tables

**Table 1:** Correlative studies between microbiota and pancreatic cancer.

Study	Specimen	No. of Samples Analyzed	Enriched Microbial Species	Notes
Fan, X. et al., 2018 [36]	Oral samples	361 PDAC, 371 healthy controls	Increased *Aggregatibacter actinomycetemcomitans* and *Porphyromonas gingivalis.* Decreased *Leptotrichia* in patients with PDAC	
Aykut et al., 2019 [37]	Tumor	13 PDAC, 5 healthy controls	Increased *Malassezia* in patients with PDAC	
Pushalkar et al., 2018 [32]	Fecal	32 PDAC, 31 healthy controls	Increased *Synergistetes*, *Proteobacteria* and *Euryarchaeota* in patients with PDAC	
Mei et al., 2018 [28]	Duodenal mucosa	14 PDAC, 14 healthy controls	Inreased *Aquabacterium*, *Rahnella*, *Oceanobacillus*, *Acinetobacter*, *Delftia*, *Massilia*, *Sphingobium* and *Deinococcus* in patients with PDAC	
Geller et al., 2017 [31]	Tumor	113 PDAC, 20 healthy controls	Increased *Pseudomonadaceae* and *Enterobacteriaceae* in patients with PDAC	Increased intratumoral gemcitabine metabolism by bacteria leading to therapy resistance
Gaiser et al., 2019 [38]	Cyst fluid from Intraductal Papillary Mucinous Neoplasm (IPMNs)	21 non-IPMN, 57 IPMN, 27 IPMN with invasive cancer	Increased *Granulicatella*, *Serratia* and *Fusobacterium.* Decreased *Methylobacterium*, *Sphingomonas* and *Propionibacterium* in patients with IPMN with high-grade dysplasia	
Kartal et al., 2022 [24]	Fecal sample	57 PDAC, 50 controls, 29 chronic pancreatitis patients	Increased *Veillonella*, *Streptococcus* *Akkermansia*	
Nagata et al., 2022 [25]	Fecal sample	43 PDAC, 235 controls	Increased *Veillonella parvula*, *Veillonella atypica*, *Streptococcus anginosus* and *Streptococcus oralis.* Decreased *Eubacterium rectale*, *F. prausnitzii* and *Ruminococcus bicirculans*	

## Data Availability

No new data were created or analyzed in this study.
